# Personal and related kinds of proximity driving collaborations: a multi-case study of Dutch nanotechnology researchers

**DOI:** 10.1186/s40064-016-3445-1

**Published:** 2016-10-07

**Authors:** Claudia Werker, Ward Ooms, Marjolein C. J. Caniëls

**Affiliations:** 1 Department of Technology, Policy and Management, Delft University of Technology, Jaffalaan 5, 2628 BX Delft, The Netherlands; 2 Faculty of Management, Science, and Technology, Open University in the Netherlands (OUNL), P.O. Box 2960, 6401 DL Heerlen, The Netherlands

## Abstract

Previous studies investigating proximity and collaboration have not clarified personal elements, such as working or communication style. Here, we show that personal proximity—close similarity in terms of personal traits and behavioral patterns—substantially affects the whole life cycle of research collaborations. We conduct a multi-case study of Dutch nanotechnology researchers. We select our interviewees through a bibliometric analysis and focus on the most central Dutch nanotechnology researchers in the global network. Our results reveal that social proximity and temporary geographical proximity have indirect effects enabling potential partners to assess their personal proximity. Sufficient levels of personal proximity often make or break the deal, provided that partners’ cognitive and organizational proximity—which are major drivers of research collaborations—suffice. Introducing personal proximity to analyze research collaborations puts previous findings on proximity dimensions’ effect on collaboration in a new perspective.

## Background

Ever since the Industrial Revolution collaborations have been driving innovation and technological change (Mowery 2009). They have substantially contributed to the creation and transfer of knowledge and innovation (e.g. Caniëls and Van den Bosch 2011; D’Este and Patel 2007; Gilsing et al. 2011). Collaborations increase the effectiveness of research processes as well as research output (Katz and Martin 1997: 15).

University researchers work with a variety of partners. When they collaborate within academia they do so to publish books, refereed journal papers and conference papers as well as to commercialize scientific insights, to produce prototypes and patents, and to apply for research grants (Jha and Welch 2010). This extended output of collaborations within academia goes together with learning effects by transferring tacit knowledge between partners (Bozeman and Corley 2004) as well as with generating more high quality knowledge (Jha and Welch 2010). When university researchers collaborate with industry partners they do not only do so in spin-off firms but also via so-called ‘academic engagement’, which captures a variety of inter-organizational collaboration mechanisms (Perkmann et al. 2013). These range from joint research projects to contract research but also involve more informal relationships between the partners. University-industry collaborations often emerge from relationships on the individual level and aim at added value for both the academic and non-academic partner (Perkmann et al. 2013).

Different kinds of proximity either enable or hamper collaborations. The results of theoretical and empirical analyses looking into proximity and collaboration suggest that geographical, organizational, institutional, cognitive and social proximity drive collaborations in various combinations and ways (e.g. Boschma 2005; Broekel and Boschma 2012; Hansen 2014a; Mattes 2012).

So far little is known about whether and how proximity on a personal level or a lack thereof affects collaborations. Yet, there is ample reason to believe that personal elements affect collaborations. Academic engagement activities center around the individual: “Both academic engagement and commercialization tend to be individually driven and pursued on a discretionary basis” (Perkmann et al. 2013: 424).

We use the concept of personal proximity to account for the personal characteristics of collaboration partners. This concept encompasses the degree of similarity in agents’ personal features, characteristics and behaviors (cf. Caniëls et al. 2014). The assumption is that the less partners differ, the more likely they will ‘click’ on a personal level. Specifically, collaborations thrive on “a mutual feeling of acceptance, appreciation and interest in each other’s ideas” (Caniëls et al. 2014: 227).

Until now, we have neither empirical insights into the effects of personal proximity on the formation, the maintenance and the output of collaboration nor an understanding of its interaction with related kinds of proximity. Investigating the influence of personal proximity on collaborations will help us to better understand the behavior of individuals and its impact on the dynamics of knowledge networks. In turn, this will inform management and policy on how to influence collaborations via personal and related kinds of proximity.

We aim to empirically investigate how personal and related kinds of proximity either enable or hamper collaborations, thereby advancing the theoretical concepts of personal and related kinds of proximity. We use two kinds of data: First, to set the scene we analyze quantitative data to understand the position of the researchers we interviewed in the worldwide nanotechnology network using a publication analysis. Second, to analyze the role of personal and related proximities for collaborations we employ qualitative data. For this purpose we interviewed nanotechnology researchers at three Dutch universities of technology.

By focusing our study on nanotechnology, we are able to investigate personal and related kinds of proximity in research collaborations in a context where these collaborations are of particular importance to the technology’s development. As we will explain, nanotechnology is in the process of moving from discovery to commercialization (Shapira et al. 2011). Thus, scientific knowledge about the technology is being transferred to industry at an ever large scale. Knowledge transfer from an academic environment to industrial science goes beyond sharing codified knowledge—e.g. through publications and patents—as it likely requires actual interaction and collaboration to overcome cognitive distances that complicate interpretation of the knowledge in codified form (e.g. Dasgupta and David 1994). Therefore, it is paramount to understand whether alternative dimensions of proximity can be used to overcome inherent cognitive distance. Furthermore, nanotechnology in particular involves scholars from various macro-disciplines (Porter and Youtie 2009), and such collaborating researchers may face considerable cognitive distance between them. Again, for the sake of nanotechnologies’ continued development, it is crucial to understand what other forms of proximity may help to overcome such distance. Individual level factors are shown to be promising levers to enable interdisciplinary collaborations that are essential to the development of nanotechnology (Van Rijnsoever and Hessels 2011). Personal proximity may constitute an important individual level factor.

Our results show that social proximity and temporary geographical proximity indirectly affect collaboration, by enabling potential partners to assess their personal proximity. Personal proximity, in turn, makes or breaks the deal in forming, maintaining, producing in, and continuing collaborations. It is shown to help partners to better exploit their organizational and cognitive proximity. In contrast, when personal proximity is lacking, this associates with detrimental performance of the collaboration and may inspire termination.

In the remainder of the paper we provide a theoretical background of the relationship between proximity and collaboration to then discuss the specific role of personal proximity in this context. We focus on the whole life cycle of collaboration in our analysis rather than just collaborations’ output. Proximity influences whether partners form a collaboration, how they work together, whether they continue a collaboration, and how productive they are. We focus on situations where agents have good abilities to assess each other’s competences and are not hampered too severely by resource limitations (e.g. own reputation/attractiveness) in selecting collaboration partners. After discussing the theoretical concept we introduce the data, motivate why we interview Dutch nanotechnology researchers, and discuss our procedure for data analysis. Subsequently, we empirically analyze the role of personal and related kinds of proximity for collaborations. After discussing the theoretical and practical contributions of our results we round our paper with a brief summary and three roads for further investigations emerging from our results.

## Theory

### State of the art: proximity affecting research collaborations

Collaboration is crucial for the exploration and exploitation of key emerging technologies, such as nanotechnology (e.g. CEC 2009). Different kinds of proximity may enable or hinder collaborations. A lack of proximity between partners can make collaborations unproductive or even impossible. Thus far, empirical and theoretical analyses suggest that various combinations of different kinds of proximity enable and foster collaborative activities.

To account for the state-of-the-art of the proximity effects on collaboration we summarize its dimensions as these have been addressed in the current literature, i.e. geographical, cognitive, institutional, organizational and social proximity (e.g. Boschma 2005; Knoben and Oerlemans 2006). For each dimension we set out distinct attributes and the level of analysis in Table 1. The table shows that different forms of proximity interactively govern collaborative behavior and output.

**Table 1:** Reification of the proximity concept

Proximities	Distinct attributes^a^	Level of analysis
Geographical	Location (pure physical distance)	Macro and meso (international/national/global/local)
Institutional	Formal and informal rules & regulations imposed by specific administrative geographical territories, such as countries and regional entities, including cultural aspects	Macro (nation/region)
Social	Embeddedness in knowledge fields, professional associations or social communities	Meso (networks)
Organizational	Organizational objectives and organization-specific formal and informal rules & regulations (including aspects of organizational culture)	Meso (organizations)
Cognitive	Knowledge areas of expertise and experience as well as reputational standing	Micro (individual)
Personal	Personal character traits, behavioural patterns, and enjoyment of one another’s company	Micro (individual)

^a^Adapted, revised and extended based on Caniëls et al. ( 2014, p. 232) and Boschma (2005, p. 71)

When push comes to shove, *geographical proximity* may not be decisive in collaborations. Rather, geographical closeness is often substituted by cognitive and organizational proximity (Capaldo and Petruzzelli 2014; Hansen 2014b), social proximity (Cassi and Plunket 2015), or temporary geographical proximity (Torre 2008). Despite the fact that geographical proximity seems to positively influence the likelihood of partner selection (Broström 2010; Hoekman et al. 2010; Ponds et al. 2007), it does not necessarily lead to output of high quality (Bercovitz and Feldman 2011; Heringa et al. 2014). This does not necessarily mean that geography is dead (Morgan 2004). For example, many studies have illustrated how geographical closeness facilitates local spillovers of knowledge as well as exploitation of local research talent (Audretsch and Feldman 1996; Broekel and Boschma 2012; Cunningham and Werker 2012). In sum, geographical proximity seems to play a role by facilitating effective and efficient collaborations, but is substitutable.

*Cognitive proximity* positively affects research collaborations. It captures partners’ similarity in terms of expertise and experience in specific knowledge fields (Boschma 2005; Knoben and Oerlemans 2006) and may also capture partners’ reputational standing (as it reflects one’s expertise in the knowledge field; Caniëls et al. 2014). Quite a number of empirical studies identify an inverse U-shaped relationship between cognitive proximity and collaborative behavior or collaboration performance (Broekel and Boschma 2012; Cunningham and Werker 2012; Huber 2012; Nooteboom et al. 2007). They support the view that potential partners are best cognitively close but not too close. On the one hand, having expertise and experience in similar knowledge areas facilitates mutual understanding of partners, thereby avoiding misunderstanding that partners from different cognitive backgrounds may encounter. On the other hand, imperfect cognitive proximity may increase the potential for innovation as long as knowledge is complementary (Bercovitz and Feldman 2011; Boschma 2005). In fact, some cognitive distance is necessary to prevent cognitive lock-in, as lock-in hampers innovation (Boschma 2005; Visser and Boschma 2004). A few studies do not find the U-shaped relationship between cognitive proximity and collaboration, with Heringa et al. (2014) finding a positive but not an inverse U-shaped relationship, and Balland (2012) finding no significant effect.

*Institutional proximity* captures similarity in humanly devised informal and formal rules and regulations that individuals adhere to in their social interactions. Informal rules include joint sets of norms and values that individuals and groups identify with as well as cultural elements supporting communication and exchange (Boschma 2005; North 1991). Formal rules consist of laws, rules, and regulations (Boschma 2005; North 1991) which may develop both on the macro-level (nations, regions, and cities) and on the meso-level (organizations or even dyadic relationships). Institutional proximity differs from organizational proximity: for institutional proximity we focus only on those rules and regulations imposed by administrative geographical entities, i.e. the macro-level. Studies addressing institutional proximity indicate that it affects collaborative behavior in a positive way. Recent findings support this claim, as institutional proximity fosters non-local collaboration (Hong and Su 2013) and eases collaborations between partners from diverse types of organizations (Ponds 2009). At the same time the absence of institutional proximity impedes collaborations. For example, it is difficult for partners from different administrative geographical areas to collaborate when they are subjected to different national legislation, e.g. regarding the conditions for research funding programs.

*Organizational proximity* captures similarity in terms of organizational goals and organizational institutions (meso-level) and serves as enabler of collective action by reducing both uncertainty and transaction costs (Boschma 2005; Caniëls et al. 2014). Potential partners are organizationally close when they are working towards similar or complementary objectives. This is the case when partners aim at the same output goals (e.g. publications, prototypes, patents, research grants) or goals with a similar time horizon. Moreover, potential partners who are organizationally different (e.g. by working for either firms, universities, or government) are subject to different institutions, i.e. different organizational structures or cultures. Academics engage with firms to pursue organizational goals that differ from those of firms (David 2004; Perkmann et al. 2013), both in time span (long term vs. short term), in terms of output (broadly, advancement of science vs. product development) and openness (public good vs. appropriation of findings). Correspondingly, organizational structures and cultures differ. Generally, theoretical and empirical analyses suggest that organizational proximity positively affects collaborations. However, findings are inconclusive with regard to the effect’s exact nature. Some find inverse U-shaped relationships while others identify indirect effects of organizational proximity (e.g. Balland 2012; Broekel and Boschma 2012; Cunningham and Werker 2012; Heringa et al. 2014).

*Social proximity* indirectly and positively shapes collaborative behavior. Partners are socially close if they are subject to the same or similar set of rules. Importantly, social rules do not stem from geographically demarcated groups. Rather, they are derived from membership of groups such as professional or sports associations, knowledge fields, and social communities. These entities connect on the basis of their shared enthusiasm or interests as well as through networks of family and friendship ties (Amin and Cohendet 2005; Caniëls et al. 2014). Hence, social proximity is the result of a joint socialization process (Boschma 2005; Caniëls et al. 2014). The concept of social proximity has emerged from Granovetter’s (1985) notion of social embeddedness building trust among individuals and reduces opportunism in social transactions. The closer partners are socially, the more they trust each other, and the less likely they are to exhibit opportunism in their behavior towards one another. The indirect and positive effect of social proximity on collaborations is acknowledged in various empirical works, although the exact nature of the effect remains hazy (Autant-Bernard et al. 2007; Balland 2012).

### Personal proximity enabling or hindering collaborations

Personal proximity captures similarities between partners with regard to “… their specific personality traits, the resulting behavioral patterns, and the degree to which they enjoy each other’s company” (Caniëls et al. 2014: 227). Similarity on the personal level emerges from individual characteristics, e.g. age, sex, and tenure (Zenger and Lawrence 1989), from traits related to the Big Five personality dimensions, e.g. extraversion, openness, agreeableness, conscientiousness, and emotional stability (Hogan and Holland 2003), as well as from the resultant behavior.

In the past, aspects of personal proximity have been discussed in three contexts: First, some analyses conflated personal proximity with social proximity (e.g. Heringa et al. 2014; Knoben and Oerlemans 2006). The two proximities are very different though. Social proximity—often measured as being directly or indirectly connected through professional, friendship or family ties—is often linked to the formation of collaborations (e.g. Cassi and Plunket 2015), because individuals tend to collaborate more with acquaintances than with complete strangers. The social proximity concept originates from studies about social embeddedness (Boschma 2005; Granovetter 1985), where it reflects the extent to which social networks of actors overlap. Hence, it reflects the *structure* of individuals’ ties. However, structural closeness in social networks does not imply that individuals would collaborate with each and every acquaintance. For instance, when seeking advice individuals are shown to prefer someone personally close, based on personal likes and dislikes (Casciaro and Lobo 2008; Yuan et al. 2014), rather than someone who is socially close (even if this person can offer more expert advice). Thus, personal proximity goes beyond the social structure of ties and focuses on the *content* of individuals’ ties.

Second, the concept of personal proximity itself was first explicitly mentioned by Schamp et al. (2004: 619) who find “personal acquaintances” to constitute an important channel for automotive suppliers to obtain timely information on planning of new models and to secure orders for those models’ parts. The closer partners are on the personal level, the more likely they are to collaborate. However, to our knowledge neither Schamp et al. (2004) nor anyone else have further elaborated upon the concept.

Third, the notion of personal proximity used here (Caniëls et al. 2014) builds on theoretical contributions to organizational psychology, specifically its principle of ‘homophily’, which poses that “similarity breeds connection” (McPherson et al. 2001: 415). Homophily affects a variety of socio-spatial relationships, such as the development of networks for discussion (Marsden 1987) and the formation of friendship ties (Verbrugge 1983). Implications of similarity on the personal level also play a role in ethical decision-making situations, where ‘psychological proximity’—involving empathy and identification with another individual on the personal level—was found to influence the moral intensity experienced when faced with ethical dilemmas (Jones 1991).

We suggest an inverse U-shaped relationship between personal proximity and collaboration. Personal proximity is likely to either enable or hinder collaborations as it works in three ways:

1. It positively affects collaborations up to a point where the similarity is too large.
2. It negatively affects collaborations in cases of too large similarity between partners.
3. It hampers collaborations when lacking.

Let us briefly illustrate these mechanisms by considering the potential effects of personal proximity on the formation, process, and outcomes of collaboration. Regarding its first effect, personal proximity is likely to trigger the selection of partners. With respect to partner selection, people prefer to tap the knowledge of persons they like (Casciaro and Lobo 2008; Yuan et al. 2014). To give an example, informal interactions between researchers and industrial firms increase both the likelihood and intensity of research collaboration (Ponomariov and Boardman 2008). From a process perspective, personal proximity eases collaboration processes, for example, because partners may share a sense of humor that enables them to appreciate and put into perspective hard but necessary critique from one another (Robert and Wilbanks 2012). Hence, it counteracts both conflicts that may hamper collaboration. Additionally, personal proximity may benefit collaborations’ outcomes. That is, partners who are personally close might produce more collaborative output. In the long run, partners who ‘click’ on the personal level collaborate on more diverse projects (Jha and Welch 2010), in other words, they produce richer outputs.

Regarding the second effect, personal proximity may also backfire on collaborations via various mechanisms. Too large similarity between partners on the personal level is particularly likely to hamper collaboration processes and outcomes. The process of collaboration may be rife of misplaced trust and immoral action, exposes both partners to risk of opportunism, and may cause blind spots. For example, evidence from organizational psychology suggests that personal proximity may lead to misplaced trust or immoral action (e.g. Burger 1981). Other research (e.g. Ingram and Morris 2007) highlights that extensive personal proximity in research collaborations may also make one vulnerable to opportunistic behavior of the other party or blind to cognitive or organizational mismatches that surface over time. Moreover, the outcomes of collaboration may suffer from the selection of suboptimal partners in terms of expertise, which may happen when there is too large personal proximity. Personal preferences may cause partners to favor collaboration with less competent partners over collaboration with more competent partners (Casciaro and Lobo 2008). Although the task at hand may still be completed when working with a less competent partner, the expertise of the less personally close partner would likely have yielded qualitatively better outputs.

Regarding the third effect, personal distance obstructs collaborator selection, complicates the process, and hampers collaborations’ output. Considering collaborator selection, in the absence of personal proximity people are less likely to seek out one another for collaborations (Casciaro and Lobo 2008; Yuan et al. 2014). They do not sufficiently trust each other as they lack the background information and experience of working with the potential partner or simply do not like the potential partner. As a consequence, they are not willing to take the risk to embark on an inherently risky research project with them. Should it, however, come to the formation of collaborative tie despite personal distance, the collaboration process is complicated. The lack of sympathy associated with personal distance may cause partners to refrain from leveraging the relevant knowledge residing in their networks (Yuan et al. 2014) as well as from sharing the knowledge necessary to complete the collaborative work. Consequently, this would also hamper the collaboration’s output.

To sum up, we propose that there is a range of personal proximity (close but not too close) that instills sufficient understanding and trust in partners enabling them to critically assess the collaboration and its progress while working together. This means that we expect an inverse U-shaped relationship between personal proximity on the one hand and the formation, process and output of collaboration on the other hand.

## Data

### Proximity affecting collaborations in nanotechnology: choosing a technology

In order to gain detailed empirical insights into how proximity in general and personal proximity in particular influences collaborations we focus on collaborations in nanotechnology. There are three reasons for our choice: First, in the recent decades nanotechnology has surfaced as a key driver of scientific and economic development. Nanotechnology has been crucial for innovation, technological change and growth in regions and countries worldwide, because its development and deployment affected other technologies and industries (Bozeman et al. 2007; CEC 2009; Salerno et al. 2008). Specifically, nanotechnology has “redefine[d] existing industries and array[ed] them in new combinations” (Bozeman et al. 2007: 807). Second, nanotechnology provides an encompassing mix of applications, including nanomedicine, nanogels, and nanocomputing devices (Youtie et al. 2008). It has shifted from discovery to commercialization (Shapira et al. 2011) and therefore requires increasingly more university–industry collaborations. Consequently, it is likely that nanotechnology-collaborations often lack organizational and social proximity. This means that potential partners may neither share goals nor professional associations. Third, collaboration between partners has been crucial for developing and deploying nanotechnology. Nanotechnology has been driving research and development crossing the borders of scientific disciplines (CEC 2009; Salerno et al. 2008), thereby shaping technologies such as information and communication technologies and biotechnology (Bozeman et al. 2007; CEC 2009). It has originated from and has linked a variety of science and engineering disciplines as revealed by the significant extent to which authors publishing on nanotechnology cite across macro-disciplines (Porter and Youtie 2009). The multidisciplinary nature of nanotechnology and the resulting diverging cognitive bases of partners coming from different disciplines and sectors often limit cognitive proximity. At the same time, partners need to be sufficiently able to understand each other to collaborate effectively and efficiently. Thus, the characteristics of nanotechnology call for collaboration enabled by proximity between partners.

Earlier investigations in the field of nanotechnology suggest that geographical, technological, social and organizational proximity affect collaborations. Cunningham and Werker (2012) found that while organizational proximity influences the output of collaborations only indirectly, geographical and technological proximity do so directly. Moreover, Autant-Bernard et al. (2007) showed that social network effects and geographical proximity mattered.

As nanotechnologies’ further advancement depends on effective integration of scientific disciplines and industrial sectors, while at the same time several dimensions of proximity may be lacking, it is relevant to investigate the relationship between personal proximity and related kinds of proximity and nanotechnology research collaborations.

### Dutch nanotechnology researchers in the global world: a social network analysis

Analyzing *Dutch* nanotechnology researchers enables us to investigate the role of personal and related kinds of proximity for collaborations in a successful and stable environment, thereby excluding major socio-economic changes and shocks that might influence our results. The Netherlands belong to the most important nanotechnology countries within the European Union (EU) (CEC 2009). When comparing Dutch nanotechnology publications (Forfas 2010) and patent applications (Miyazaki and Islam 2007) with those of other EU countries, the Netherlands came in fourth, with Germany being first, and the U.K. and France were second and third respectively. When looking at the worldwide output of nanotechnology publications the Netherlands are an important player (Cunningham and Werker 2011), as they are ranked 8th in the list of most productive countries worldwide—accounting for number of publications per million citizens. The Dutch environment even served as a benchmark for others, such as Ireland (Forfas 2010).

Dutch nanotechnology researchers are close to the core of the global nanotechnology network. To illustrate the relevance of Dutch nanotechnology research we analyzed the worldwide network of nanotechnology researchers by conducting a bibliometric analysis based on publication data from the Web of Science databases using the updated lexical search query by Arora et al. (2013). The overall nanotechnology network for the 2011–13 period consists of approximately 637,902 researchers who form 23,447 connected communities. The largest connected component in the network consists of more than 85 % of the overall network, a community of 543,560 researchers. From this largest component we extracted the top 200 Dutch nanotechnology researchers based on their Eigenvector centrality, which indicates the power of an individual in the overall network (Bonacich 1987, 2007). Our results show that Dutch nanotechnology researchers hold quite a central position (see Table 2 and Appendix for selected Dutch researchers’ network statistics relative to those of the global top and bottom researchers). Dutch nanotechnology researchers are in a position of similar or better centrality than the most central researchers globally. This holds in terms of closeness centrality, measuring the path length to all other nodes in the network (Takes and Kosters 2013), and also in terms of eccentricity, measuring the maximum distance from one node to all other nodes in the network (Takes and Kosters 2013). In terms of Eigenvector centrality and degree centrality, measuring the number of direct ties (Takes and Kosters 2013), the Dutch nanotechnology researchers are in a weaker position than the global top 5, but a substantially better position than the global bottom 5.

**Table 2:** Interviewees and their centrality in the global nanotechnology research network

Interviewee^a, b, c^	Eigenvector centrality	Closeness centrality	Degree centrality	Eccentricity
TUD1A	1.39 × 10^−6^	0.18	1.06 × 10^−4^	14
TUD1B	5.98 × 10^−6^	0.19	1.16 × 10^−4^	13
TUE1A	7.69 × 10^−7^	0.17	5.70 × 10^−5^	15
TUE1B	5.31 × 10^−6^	0.19	8.46 × 10^−5^	14
UT1A	2.26 × 10^−6^	0.18	7.73 × 10^−5^	14
UT1B	7.32 × 10^−7^	0.17	3.86 × 10^−5^	14
TUD2A	3.76 × 10^−7^	0.17	6.07 × 10^−5^	14
TUD2B	1.77 × 10^−6^	0.17	1.16 × 10^−4^	15
TUE2A	6.75 × 10^−6^	0.19	1.71 × 10^−4^	13
TUE2B	7.68 × 10^−6^	0.19	1.27 × 10^−4^	14
UT2A	6.03 × 10^−7^	0.17	1.12 × 10^−4^	14
UT2B	5.61 × 10^−6^	0.20	1.31 × 10^−4^	14
TUD3A	1.29 × 10^−10^	0.13	7.36 × 10^−6^	16
TUD3B	1.02 × 10^−6^	0.18	1.07 × 10^−4^	14
TUE3A	2.63 × 10^−6^	0.19	1.55 × 10^−4^	15
TUE3B	2.34 × 10^−6^	0.18	1.32 × 10^−4^	14
UT3A	2.36 × 10^−5^	0.17	5.89 × 10^−5^	14
UT3B	1.39 × 10^−3^	0.18	1.18 × 10^−4^	15

^a^TUD is Delft University of Technology, TUE is Eindhoven University of Technology, and UT is University of Twente

^b^The research orientation is indicated by the following digits: 1 (pure basic), 2 (use-inspired) and 3 (pure applied)

^c^A and B indicate the two different interviewees in every category

**Table 3:** Evidence and interpretation of relationship between cognitive and organizational proximity and collaborations

Relationship	Illustrative evidence and interpretation		
Cognitive proximity as a motive for collaborator selection	“It is a joint programme between him and (*Collaborator’s name*), that’s the mathematician, and myself (*computational catalysis*) on complexity science issues with neurons in the brain.” *(Interviewee TUE1A*-*1)* *Interpretation:* Mathematics and computational catalysis intersect in complexity sciences	“This was a group that had an extensive experience in this area. Working in a medical environment. There are research labs in the university but we wanted biology and medicine. This group was the best fit for the things we wanted to do.” *(Interviewee TUD3A*-*1)* *Interpretation:* Selected a collaborator from a group with experience in biology (like the interviewee) as well as in its application in medicine	“(*Collaborator’s name*) is a professor in my field in (*City’s name*) and is, let me say, the young star. He came to the Netherlands, he is from (*City’s name*), in 2001 or 2002 or so. And I consider him kind of my…he has a kind of position I used to have in the past in the Dutch catalysis scene. So he also participates in high level discussion with the government on scientific issues, industry is involved. He is a great scientist, also got a Spinoza grant.” *(Interviewee TUE1A*-*2)* *Interpretation:* Adjacent or similar reputational standing within the knowledge field
	“It was good fun for once, running the analyses I thought a few things were interesting, other things were not really down my alley. […] we wouldn’t initiate a follow-up project. (*When asked whether the interviewee avoided follow*-*up because of the cognitive content:*) Yes, because of content, because other than that he was a nice fellow to talk to. Just different methods, different possibilities, and other perspectives.” *(Interviewee TUD2B*-*1)* *Interpretation:* Experienced cognitive distance causes interviewee to refrain from collaboration	“No, it was put to halt for the time being, because we now focus on materials instead.” *(Interviewee TUE3B*-*1)* *Interpretation:* A change in expertise of the researcher’s group created cognitive distance and, thus, led to (temporary) withdrawal from the collaboration	
Organizational proximity as a motive for collaborator selection	“This is a European consortium. There is a consortium agreement which says that everyone is the owner of its own development, but as soon as you co-develop things then you have to agree on what you do with the results. That works very well together. It is a little bit easier in an institute like (*European consortium involving universities and public research organization*) than with a company. Because a company is really focussed on intellectual property. Also these institutes are a little less secretive. It is a little easier to cooperate.” *(Interviewee TUE2A*-*1)* *Interpretation:* Organizational structures and goals of universities and PROs integrate more easily than those of universities and firms	“Well, because of this we have been able to publish a couple of very nice papers and conduct truly exciting scientific research. In my view, the sum of the collaboration is more than either of us could ever do individually.” *(Interviewee UT1A*-*1)* *Interpretation:* The shared organizational objective of wanting to publish good quality papers and be on the frontier of science is what motivates these collaborators to work together	“What is very important is that should also realize that there should be added-value for both sides. You cannot start collaboration only out of your interest, it won’t work. So you have to realize what the added-value is for the other.” *(Interviewee UT2A*-*1)* *Interpretation:* Importance of added-value
Cognitive and organizational proximity driving collaborations	“[*Collaborator’s name*] has been extremely instrumental to confirm that a couple of times […] On the other hand, it turns out that this is not a collaboration where we have something and they merely confirm it. They have their own questions as well, where their technique indicates some unique stuff is going on but the materials are unsuited to really shed light on the phenomenon. So, we can basically tweak the properties of the material in our laboratory—tinker with the molecule, if you will—to expose this unique property. In that way, we have found a really lovely collaboration, because well, it is not only of my interest that he does measurements […] but it is also interesting for him and his group to seek collaborations with me and my people. We can offer them materials that are unique to the world.” *(Interviewee UT1A*-*2)* *Interpretation:* Cognitive proximity as one collaborator is able to do measurements and the other is able to alter properties of one material. Organizational proximity because the activities by help the other’s progress and vice versa	“I would say it has partly to do with the content. We were building certain (*high technology prototypes*) and then you are glad when somebody comes along and says he needs it. Because the (*technology*) on its own is interesting in terms of physics, but it is a little bit academic. There is always an extra satisfaction if you see that other people like what you do for non-academic reasons. For example, for potential applications and solving other types of problems that I have never thought about. So that was content-based attraction.” *(Interviewee UT3A*-*1)* *Interpretation*: Content-based attraction (cognitive proximity) and interdependent goals (academic and non-academic goals are complementary; organizational proximity)	“My story was that I knew that exotic field and I knew that I didn’t have clue how to work on it and then I heard his name […] So that led to informal chat and then a sort of flash that it will be something good for the project. I saw a sort of connection between what I knew and sort of an opportunity.” *(Interviewee TUD2A*-*1)* *Interpretation:* Cognitive proximity as one has basic knowledge of a field and the other somewhat more advanced. Organizational proximity because of the perceived opportunity to collaborate to the benefit of an ongoing project

**Table 4:** Evidence and interpretation of moderation by personal proximity

Relationship	Illustrative evidence and interpretation		
Indirect effect of personal proximity	“Knowing each other personally helps to make the collaboration go smoother and better. There are also some collaborations where we did not know each other that well on beforehand, but you were put together by coincidence, and find out that it works well.” *(Interviewee TUE2A*-*2)* *Interpretation:* Personal proximity enhances the collaborative process. Also when it develops during the course of the collaboration	“…you can make clear what he does, how it affects you and why you are not liking that. So it becomes a boundary condition for him and he can adjust himself. […] I had one guy who also understood this very well […] but if that is not the case I simply do not want to have the collaboration.” *(Interviewee TUD2A*-*2)* *Interpretation:* Mutual understanding about acceptable behavioural patterns of one another	“The key element is working with people whom you know, trust and respect.” *(Interviewee TUD3A*-*2)* *Interpretation:* Being familiar with the collaborator and respecting the person is important to the collaborative process
Personal proximity moderating the effect of cognitive and/or organizational proximity	“I think the quality of output has to do with the real scientific expertise of the other. I think it is very important to have a high degree of personal understanding, because then you can solve all kinds of problems. But for high quality output you need the expertise and then when it becomes important to collaborate with somebody—yeah, simply said—who you do not like, or don’t like so much, but you know what that person does is really high quality…then you had better listen to him content wise.” *(Interviewee UT3A*-*2)* *Interpretation*: Illustrates how cognitive proximity has a direct effect and how personal proximity may indirectly enhance its impact	“You take on a responsibility. It says nowhere in the responsibility: “Oh, you don’t have to do that because you don’t like the people.” No, that is not part of the responsibility, the responsibility is to get the job done. You accept the funds. Sometimes you discover after a year that the chemistry is not great. But that’s okay. Everybody is professional. You behave like a professional and you get the work done.” *(Interviewee TUD3A*-*3)* *Interpretation:* Goals are interdependent because of requirements set by funding organization. Yet, personal proximity may hamper the collaborative process, leading to eventual discontinuation once goals have been reached	“We were enthusiastic about the options on both sides. There was a good personal connection which is based on trust. He grows things that not many people in the world grow, so he makes special materials. I have a special tool. So together we can do something that is, again, rather special.” *(Interviewee TUE3A*-*1)* *Interpretation:* Collaborators depend on their cognitive complementarities to attain a special goal. Their collaboration is possible because they ‘click’ on the personal level
	“We never had problems in giving feedback to each other. But of course it is always in a way that you respect each other. That’s very important. […] If you do not respect each other, you cannot collaborate. Collaboration is a win–win situation on both sides […] it should be beneficial for both partners otherwise it is not a real and successful collaboration.” *(Interviewee UT2A*-*2)* *Interpretation:* Interviewee identifies most with collaborators who are respectful in their feedback. Hence, personal proximity may enable to exploit potential win–win situations where organizational goals coincide	“The first would still be technical closeness, but it should be a bit dissimilar. It should not be exactly the same. The first thing is that you need to be able to do something together. For me it is not much use to work with somebody who is into philosophy or high energy particles, because these are not my areas. I will not collaborate with them, even if they are my biggest friends. So, having some kind of mutual interest is important. I think the second thing is that for me it is important to know the person a little bit. Of course I can collaborate with people that I don’t know that well, but usually it helps to know a person.” *(Interviewee TUE2A*-*3)* *Interpretation:* Cognitive proximity is required up to a certain extent. Personal proximity may increase the likelihood of collaborator selection and help collaborators to exploit cognitive proximity	“I was tipped by a colleague to talk to him, because he was a theorist. He made the system on which we were working experimentally. So in that conference we had a discussion, we arranged the meeting. It was very clear that we were more complete together: I had the data he wanted, he had the calculation I needed. So there was a perfect match. It turned out that there were more levels of connection, different subjects on which we were working here. He became a very valuable colleague for me. And he is also a personal friend. We visit families over. (*When asked about repeated collaborations:*) Absolutely. Since 2003, we meet at least 4 times a year either in the US or here. Apart from that we meet at conferences. He was here 2 weeks ago. [*Later in this interview:*] Just to be friends is not enough. I try to consider my collaborators as scientific friends.” *(Interviewee TUE3A*-*2)* *Interpretation:* Cognitive proximity and organizational proximity are the fundament of this collaboration. However, personal closeness has developed and helped to sustain the collaboration
	“[*After having explained the cognitive complementarity found in a particular collaboration:*] It often boils down to having a bit of a personal click, as into what extent it is fun to collaborate, and of course posing original ideas, that helps as well.” *(Interviewee TUD2B*-*2)* *Interpretation*: Show that the interviewee values being able to get along with cognitively close collaborators	“He wanted to collaborate with me because I had developed this special technique […] and I wanted to collaborate with him because he was well-informed about crystals theoretically. […] The click I have with [*Collaborator’s name*] is completely different from the one I have with (*Names of two other collaborators*) though. The ones from (*University’s name*) are really my scientific parents or fathers. (*Collaborator’s name*) is a friend of roughly the same age.” *(Interviewee UT2B*-*1)* *Interpretation:* Motive to collaborate is cognitive and organizational in nature, personal proximity (derived from different sources) comes into play as well	“[*When asked for his motives for engaging in a particular collaboration:*] That is content, mutual expertise, mutual facilities that may be complementary. Somewhat of an overlap in research interests and also a little of a personal click. […] Well, with some people you get along well on the personal as well as the scientific level and I am more likely to go and talk to those people and see where that takes us.” (*Interviewee TUE1B*-*1*) *Interpretation:* Cognitive and organizational proximity are requirements, personal proximity then guides the choice between alternatives

**Table 5:** Evidence and interpretation of personal proximity as a mediator of social proximity and temporary geographical proximity

Relationship	Illustrative evidence and interpretation		
Personal proximity mediating the effect of social proximity on collaborations	“Knowing the person personally well plays an important role, although not always. Professional relationships, people you meet in conferences continually and you discover that you have common interests, expertise that are complementary so that you can do more together than you could do separately and then one thing leads to another. Then you start doing collaborative research. It has never occurred to me: “Hey, I have an idea and I need a chemist, I have got to go and find a chemist!” That has never happened to me. […] Maybe it is interaction with the people which stimulates me to think of collaborative projects and then I know immediately who the person is going to be.” *(Interviewee TUD3A*-*4)* *Interpretation:* Social proximity allows one to familiarize with potential collaborators	“Yes, [*Collaborator’s name*] has joined the team at [*Name of a university spin*-*off firm*] as a researcher when I was still part-time project leader at the place. I know him well on the personal level. We were on the same hockey team for five years or so.” *(Interviewee TUD1A*-*1)* *Interpretation:* Social proximity (working at the same spin-off firm and sharing membership of a hockey team) feeds personal closeness, which in turn led the interviewee to select him as a collaborator later on from an array of cognitively close potential collaborators	“There is another one, [*Collaborator’s name*], from [*Large high*-*technology firm’s name*]. […] He works in [*Large high*-*technology firm’s name*] and I also worked there, so I know him very well.” *(Interviewee UT2A*-*3)* *Interpretation*: Professionals association in the past—sharing a previous employer—enabled the collaborators to develop a close personal relationship
Personal proximity mediating the effect of temporary geographical proximity on collaborations	“What is needed is that you have the opportunity to meet and have some dinner or so together. I like to do that. Very often, if I meet them, I like to have dinner with them. Not with 10 people, but with 2 or 3, and then we talk. That’s what I like. Or lunch, or that kind of thing. But you can also sit in the train for a few hours.” *(Interviewee TUE1A*-*3)* *Interpretation:* Temporary geographic proximity as a way to assess personal fit with potential collaborators	“Because of a [*Funding organization’s name*] grant through which I visited the Institute of [*Specialization*] in [*City X*] and I saw him as an eager guy who wanted to move ahead. That was one observation. […] I had told this person *[colleague]* when he still worked in (*City Y*) that we needed for our project [*specialization*] engineers and I told him that if you really want (*specialization*) engineers, we should go to (*City X*), because in The Netherlands they are no longer trained at a sufficiently high level. And that’s how *(collaborator’s name)* came to (*City Y)*.” *(Interviewee TUD2A*-*3)* *Interpretation:* A visit abroad created temporary geographical proximity that enabled the interviewee to judge the personality of this potential collaborator	“I was there a couple of times from a consultancy perspective really, twice to be precise, and we extended our collaborative relationship when they also came to visit here. One thing led to the other…” *(Interviewee TUE2B*-*1)* *Interpretation:* Brief visits serve as a prelude to more significant collaborations

### Nanotechnology researchers at three Dutch Universities of Technology: sampling, interviewing, and analyzing

To grasp the role of personal and related kinds of proximities for collaborations of Dutch nanotechnology researchers we adopt a multiple-case design (Yin 2009). We focus on researchers working at the three Dutch universities of technology, i.e. Delft University of Technology, Eindhoven University of Technology, and the University of Twente.^1^ We proceeded in three steps: first, we identified and selected interviewees based on theoretical arguments and aided by the bibliometric analysis of the global network of nanotechnology researchers. Second, we conducted the interviews with the selected researchers, discussing their collaborations in detail. Third, we analyzed the interview data.

In a first step we theoretically sampled our interviewees (Eisenhardt 1989). We used a matched-pairs approach (Fromhold-Eisebith et al. 2014), which allows us to create pairs of researchers forming theoretically contrasting cases in terms of research orientation. We apply this sampling strategy because research orientation may affect the nature of research collaborations—i.e. collaborations have different types of goals and likely deal with different sorts of knowledge and partners accordingly (Hessels and Van Lente 2008; Nooteboom et al. 2007)—and therefore the role of personal proximity may also vary across collaborations of these researchers. To assess academics’ research orientation we adopted Stokes’ (1997) two-dimensional model characterizing research orientation based on the degree to which their research is motivated by (1) a quest for fundamental understanding and/or (2) considerations of use (following Ooms et al. 2015). The four resultant research orientations are: pure basic (Bohr quadrant), use-inspired (Pasteur quadrant), pure applied (Edison quadrant) and low overall research orientation. We sampled interviewees accordingly, disregarding the “low overall” quadrant assuming that every full professor in our sample is inherently oriented towards research. In doing so, we recognize that researchers at universities likely always consider fundamental issues, but may still predominantly pursue pure applied research (i.e. nanotechnology researchers targeting journals focused on specific applications, generating patents, or joining spin-offs). To establish interviewees’ research orientation within nanotechnology we followed Arora et al. (2013) by distinguishing knowledge fields of nanotechnology that are of a fundamental nature from those that are more application-oriented. By using Dutch university and faculty web pages with information on departments and individual researchers as well as personal web pages of researchers (studying publication overviews, professional biographies, funding sources, and press releases) we were able to identify full professors working on nanotechnology in the Bohr, Pasteur and Edison quadrants. Our sample consists of eighteen full professors. They are employed at the three Dutch universities of technology and belong to the top 200 Dutch nanotechnology researchers as identified in the bibliometric analysis (see previous section). Two researchers of each university belong to one of the three research orientations (see Table 2).

In a second step we carried out the interviews from December 2012 to February 2015. Eighteen semi-structured interviews were conducted in two rounds (in order to run a preliminary analysis, see below) with the interviewees listed in Table 2. The interview guide was inspired by our theoretical framework as to contribute to the internal validity of our study. Prior to data collection, three pilot interviews were conducted to test and improve the interview protocol. Each of the eighteen interviews covered 4–6 collaborations in detail and interviewees discussed relevant aspects of their collaborations such as partner selection and the collaboration’s activities, output and continuation. Different dimensions of proximity surfaced during these discussions. Interviewees were asked to name partners from academia as well as industry. Many interviewees provided us with handwritten lists of their partners during the interviews. We kept a case study database using the MaxQDA 11 software tool to contribute to our study’s reliability (Gibbert et al. 2008). We logged and added all interview transcripts and notes to the case study database. The average interview duration was 1 h.

In a third step we combined a deductive and an inductive coding strategy. During the deductive coding process we used codes derived from our theoretical framework and, thus, directly related to the attributes associated with different types of proximity in Table 1 (Miles et al. 2014). Additionally, we inductively created codes to capture the type of output of collaborations and interviewees’ positive or negative feelings regarding both the collaborative process and collaboration output. During the coding process we attached comments to coded segments to log the rationale for certain analytical decisions or register interpretations in more detail. Subsequently, we summarized coded segments per code per case to complete within-case analyses. Results across cases were contrasted, for example, by conducting an analysis of the relationships between codes. Specifically, we looked into the co-occurrence of codes to obtain indications for possible patterns in the data and then returned to the empirical data to understand the nature of these patterns. To establish construct validity and transparency about coding decisions we provide snippets of our interpretations with the data presented throughout the next section (following the example of Zott and Huy 2007). In the end, we compared the results of the final data analysis of eighteen interviews to the preliminary analysis of the first set of nine interviews. As the results did not change we concluded that we reached saturation.

## Personal and related kinds of proximity affecting collaborations: results from a multi-case study

The results of our multiple-case study of research collaborations by Dutch nanotechnology researchers are set out in the following sections. We describe the role personal and related kinds of proximity (see “Theory” section) play in the formation of collaborations as well as in the process and outcomes of collaborations.

In our interviews on proximity and collaboration we could not detect any systematic differences between research orientations. All researchers seem to assess and decide based on the various kinds of proximity in similar ways.

In the following we often refer to illustrative empirical data. Many illustrative quotes are presented in Tables 3, 4 and 5. Whenever illustrative data is included in one of these tables, the respective quote can be identified by the number added behind the interviewee ID, always preceded by a hyphen. In some cases, we offer more elaborate descriptions of data in the text and therefore do not include these particular quotes in our tables.

### Personal proximity supporting cognitive and organizational proximity

Our results indicate that personal proximity comes into play as soon as sufficient organizational and cognitive proximity between partners makes collaboration worthwhile. Both cognitive proximity and organizational proximity are important enablers or barriers for researchers’ collaborative activities, thereby directly affecting the formation of collaborative ties and their output (Table 3).

*Cognitive proximity* encompasses useful matches of adjacent but distinct knowledge fields (TUE1A-1). Cognitively close partners are sought for in terms of overlap or complementarity in expertise or experience. Moreover, cognitive proximity can stimulate collaborations when partners work in similar knowledge fields but have different orientations (UT3A-1). This type of cognitive proximity often drives academic engagement activities, where researchers with a rather fundamental orientation engage in collaborations with their counterparts at industrial firms who have an interest in the application of technologies.

Cognitive proximity includes reputation as well as experience and influence in the scientific community. The suggestion that reputational standing may also be weighed when determining cognitive proximity for collaborations is confirmed in the empirical data. Interviewees often work with partners of either a somewhat higher reputational standing, with the motive to benefit from this particularly experienced partner, or with partners whose reputation has not yet developed to their own level, in order to help these partners to grow their career. TUE1A-2, TUD1B and others illustrate situations in which reputational standing affected collaborative choices. Their partners are sought for their influence within the field or to establish mentoring relationships given their growth potential within the interviewee’s field.

Our findings support the results of former quantitative studies that partners should be cognitively close but not too close (e.g. Broekel and Boschma 2012; Cunningham and Werker 2012). In many interviews we find evidence illustrating that a lack of cognitive proximity hinders collaborations (interviewees TUD2B-1 and TUE3B-1). At the same time evidence suggests that partners who are too cognitively close cannot successfully collaborate. In some cases where researchers within the same organizations are too cognitively close they refrain from collaboration. For example, throughout the interview with TUD2A we learned that collaborators within his organization who work in a particular nanotechnology niche are unable and unwilling to see potentially interesting opportunities for collaboration outside of the scope of their own area, at the expense of output quality. Hence, perfect cognitive proximity is deemed undesirable in research collaborations, as some distance is required to prevent lock-in.

*Organizational proximity* is composed of two dimensions, namely similarity or complementarity in terms of (1) organization-specific institutions (i.e., rules, regulations, and cultural aspects) and (2) organizational objectives. First, when partners work for similar types of organizations they are subject to similar organizational rules, regulations and cultures. Consequently, collaborations between academic partners are easier to manage because of organizational closeness. In contrast, in cases where the interviewees collaborate with partners from industry we observe difficulties because of limited organizational proximity (see TUE2A-1 in Table 3). Second, organizational proximity drives research collaborations when partners strive to attain either similar goals or goals that are complementary in nature. Interviewees emphasize this dimension of organizational proximity more regularly than the previous one. Here, they clearly use collaboration as a means to an end which indicates that it is a vehicle for the partners to achieve certain goals. UT2A-1 points out that collaborations have to create “added-value” for both parties. Similarly, TUE1B emphasizes: “You try to generate added-value together and when you accomplish this you’re both satisfied.”

Our results indicate that *a combination of cognitive and organizational proximity* strongly supports the formation and output of collaborations. Specifically, organizational proximity in terms of complementary goals often comes with cognitive proximity. TUE3A-1 describes a collaboration in which cognitive proximity helped partners to attain a “rather special” goal by combining knowledge about “special materials” and knowledge about “tools” (see Table 4). For choosing the right partner UT1A-2 explicitly points to elements of organizational proximity (“has been extremely instrumental” and “it is not only of my interest that he does measurements […] but it is also interesting for him and his group”) and cognitive proximity (one partner supplies and tweaks the materials and the other has the ability to measure their properties). That organizational and cognitive proximity work hand in hand is supported by the fact that one hundred eleven of the coded segments in our analysis co-occur at codes for organizational and cognitive proximity. This indicates that interviewees often refer to the two dimensions in relation to one another (for more examples see Table 3).

*Personal proximity* can be a dealmaker of collaborations. It is illustrative that, after discussing his collaborations in great detail, TUD3A-2 emphasized the essence of his collaborations as “to know, trust and respect collaborators.” Interviewees take personal character traits of their potential partners into account when starting a collaboration (Table 4). For example, TUD2A-3 needed a specialized engineer to fulfil a project’s objectives (organizational proximity) and acknowledged that the right expertise and experience (cognitive proximity) was only to be found amongst researchers trained at a specific organization in another country (Table 5). However, while many could have fitted the requirements in terms of organizational and cognitive proximity, he eventually chose a partner with certain character traits, i.e. eagerness and ambition, because these mirrored his own character and motivation. Personal proximity was decisive for picking this particular partner. Table 4 presents various segments supporting this take on personal proximity (e.g. UT2A-2, TUE2A-3, TUE3A-2, TUD2B-2, UT2B-1 and TUE1B-1).

The right degree of personal proximity leads partners to enjoy each other’s company, often referred to as a ‘click’ between individuals. Interviewees regard of this ‘click’ as a fundamental building block of thriving collaborations (e.g. TUE2A-2 and TUE3A-2). When partners do not enjoy each other’s company, they lack positive reinforcement of the collaboration by personal proximity. A lack of mutual respect (e.g. TUD2A-2) or differences of working style (e.g. UT1B) can be true deal breakers for collaborations.

A lack of personal proximity does not necessarily break the deal in cases where opportunities created by cognitive and/or organizational proximity outweigh the absence of personal proximity. When expertise is attainable that enables one to realize individual or organizational objectives, many interviewees seem to be willing to set aside personal issues at least for a while. UT3A-2 refers to the role of personal proximity as a fundament to settle issues in collaborative processes. Yet, he carefully illustrates that cognitive proximity and organizational proximity can compensate for a lack of ‘clicking’. Moreover, when organizational objectives are of considerable strategic value, organizational proximity may also limit the individual’s control over formation of collaborative ties and, hence, affect the role personal proximity plays in collaborations (TUE2A-3). In any case, whether potential partners are able and willing to set aside personal distance and ‘disclicks’ seems to depend on their characters. While UT3A-2 tolerates personal distance and ‘disclicks’ to some degree, TUD2A-2 and TUE3A-2 are less willing to do so.

Personal proximity changes over time and so does its role in collaborations. While partners may conclude that they sufficiently click to embark on the collaboration based on an initial assessment, specific experiences make them change their minds in the course of collaborations. When partners feel that their expectations about each other’s personal traits are not working out they may decide to restrict or even terminate their collaboration. For example, potential partners may initially feel that they share their take on respecting the others’ work or their take on punctuality. However, they might later find out that this is not the case. To give three illustrative examples: First, UT1B ascribes failure and termination of a collaboration to his partner’s exertion of control and lack of perseverance, both of which he takes as signs of distrust and disrespect for his competencies. Second, TUE2A expresses that his collaboration was substantially hampered by different takes on punctuality. Third, TUD2B disapproves of the ignorance and neglect a former partner shows for his talent and potential. Violating personal proximity in this way may not always lead to immediate termination of the collaboration, but it will affect the decision to continue once initial goals are attained (TUD3A-3).

To sum up, our qualitative findings show the role personal proximity plays in the life cycle of collaborations given that partners are sufficiently cognitively and organizationally close.

### Personal proximity mediating social and temporary geographical proximity

Our results suggest that former analyses have conflated personal with social or temporary geographical proximity.

For *social proximity* our results show that socially close potential partners can assess whether or not they are sufficiently personally close. Many times the interviewees describe encounters with socially close individuals as the setting in which they were able to determine whether they would click with the other party. Hence, personal proximity mediates the effect of social proximity on research collaborations (see Table 5). TUD3A-4 indicates that his familiarity with an individual on the personal level is often retraceable to professional relationships—e.g. social proximity via conferences. Along the same lines, social proximity between UT2A-3 and his partner emerged from their shared employment history, as both are embedded in the network of (former) employees of a large high-technology firm and have developed a personal relationship because of this. However, social proximity is not established through professional social communities only. For example, TUD1A-1 selected a partner with whom he shared membership of a recreational sports team. In the sports team, they “clicked”. Independent of its source, social proximity serves as a mechanism to assess, develop and maintain personal proximity that can be crucial in collaborations.

A similar argument holds for *temporary geographical proximity*. As often pointed out, geographically co-located partners can have more intense knowledge exchange than partners located far apart, as they can exchange knowledge face-to-face. With our findings we support the results of previous research (Capaldo and Petruzzelli 2014; Hansen 2014b) that geographical proximity is not a crucial predictor of partner selection. Rather, cognitive and organizational proximity outweigh the convenience of being in the same location. However, ‘temporary geographical proximity’, i.e. partners co-locating for a limited period of time (e.g. Rychen and Zimmermann 2008; Torre 2008), turns out to be important for collaborations. Face-to-face interactions for short periods of time ease knowledge transfer as they enable potential partners to assess whether or not they ‘click’. For example, TUD2A-3 indicates that temporary geographical proximity was decisive in recognizing desirable character traits in a potential partner—in other words, to assess personal proximity—and thereby indirectly guided the selection of his partner.

Together, social proximity and temporary geographical proximity enable potential partners to judge their personal proximity and see whether or not they ‘click’. For example, interviewees TUE2B-1 and TUE1A-3 describe how low-key or serendipitous social encounters, where they were temporarily geographically close, inspired potential partners to seek more intense collaboration at a distance. Ultimately, these collaborations grew to be important.

In sum, by including personal proximity we get a better picture of how the different kinds of proximity interact. According to our findings both social and temporary geographical proximity merely affect collaborations by enabling potential partners to assess their personal proximity. That is, social and temporary geographic proximity allows partners to find out whether or not they click adequately to collaborate, provided that they are also cognitively and organizationally close.

## Implications

**Fig. 1:**
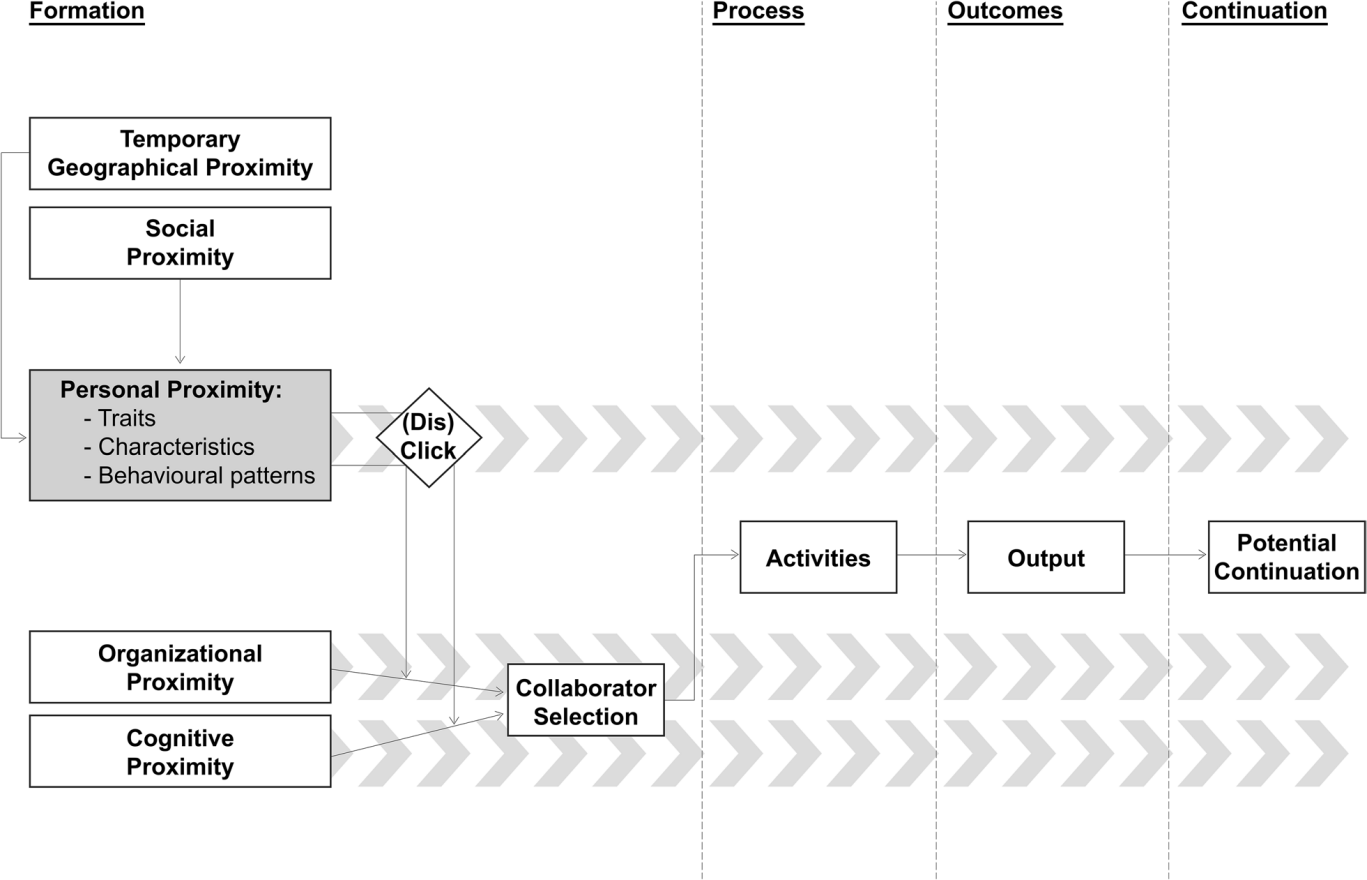
Dimensions of proximity affecting collaboration formation, process, outcomes and continuation

We find ample evidence to suggest that personal proximity affects the formation, maintenance and output of collaborations. Here, we focus on the three theoretical contributions stemming from our empirical findings (Fig. 1). The first and second contribution that we discuss hold some important implications for the proximity literature in general, while the third contribution revisits our theoretical propositions about the role of personal proximity (see “Personal proximity enabling or hindering collaborations” section).

We show the influence of personal and related kinds of proximity on collaborations (Fig. 1). We empirically analyze the relationship between personal proximity and collaborations. In doing so, we provide a more detailed picture on how the different kinds of proximity interrelate and/or substitute each other.

Our first contribution concerns the role of social proximity and geographical proximity in collaborations and the formation thereof in particular. While our results regarding organizational and cognitive proximity are in line with previous studies, they shed a different light on the relationship between social and geographical proximity on the one hand and collaborations on the other hand. Rather than directly affecting collaborations, social proximity and temporary geographical proximity facilitate the development of personal proximity, and thereby affect collaboration (Fig. 1). Potential partners can explore whether or not they sufficiently ‘click’ to collaborate successfully. To date, empirical findings on the relationship between social proximity and collaborations have been blurry at best as they did not manage to explain the exact nature of this relationship (e.g. Autant-Bernard et al. 2007; Balland 2012). Our results imply that personal proximity mediates the relationship between social proximity and collaboration. Using Granovetter’s (1985) words, “social embeddedness” allows to run reconnaissance for likeable or personally close collaborators. Researchers use their membership of professional associations, visits to regular conferences, and the network of their field of knowledge as a pool of potential partners. Thereby, our findings hint at why Cassi and Plunket (2015) find social proximity to substitute for organizational proximity and geographical proximity over time. In similar ways geographical proximity does not necessarily affect collaboration directly. At the same time, temporary geographical proximity surely affects collaboration indirectly, mediated by personal proximity. Our results are in line with former findings on the firm or cluster level (Bathelt and Schuldt 2008). Specifically, we extend Torre’s (2008) original hypothesis by proposing that temporary geographical proximity does not directly inspire collaboration, but rather enhances personal proximity and thereby positively affects collaborations.

Our second contribution is that our findings support and move forward recent discussions about the dynamic co-evolution of various kinds of proximities (Balland et al. 2015; Huber 2012). Research collaborations in our study are continued and intense interactions between two individuals. Our findings are in line with the assumption that dynamics of co-evolution are apparent in the collaborative interactions of this sort, more than in those of a shorter, less intense nature (Balland et al. 2015). In our study, personal proximity remains important throughout the whole life cycle of a collaboration as partners get to know each other better over the course of their collaboration. Thus, its impact is not limited to partner selection, but extends to joint activities, output, and decisions regarding continuation. The same considerations hold for organizational and cognitive proximity. The dynamic co-evolution we find for personal, organizational and cognitive proximity is depicted in Fig. 1 by the large grey arrow heads in the background originating from those dimensions of proximity.

Importantly, our results contradict some concerns voiced in studies that address the dynamic co-evolution of proximities to date (e.g. Balland et al. 2015), in which it is argued that intense and prolonged knowledge networking is likely to increase all sorts of proximity. That is, intense knowledge networking is assumed to spark convergence of knowledge bases (increase of cognitive proximity), social networks (increase of social proximity), and so on, leading to ‘excess’ proximity and thereby hampering collaborations’ output because of the resulting cognitive lock-ins and ignorance towards potential new partners (Boschma 2005). Instead, our results show that intense interactions do not necessarily lead to convergence on the personal dimension of proximity. Getting to know one another better, does not equate getting to like one another better. In fact, divergence may occur, as intense interaction is shown to expose traits and behaviors of partners that violate the initial perception of personal proximity and, thus, render collaboration unproductive and may cause termination of these otherwise unproductive ties. Considering that our results show how personal proximity may either decrease or increase over time, we question whether it is right to assume that other kinds of proximity will only converge as time passes. Hence, while proximities co-evolve over time, our multiple case study shows that sometimes this may also mean that proximity decreases over time. Nevertheless, the question of what to do to overcome situations in which convergence does occur remains.

Our third contribution lies in the advancement and understanding of the personal proximity concept. Our rich empirical data enables us to further refine and demarcate earlier conceptions of personal proximity (e.g. Caniëls et al. 2014; Schamp et al. 2004) by distinguishing between personal proximity on the one hand and ‘clicks’ on the other hand. We show that personal proximity entails more than just personal acquaintance as defined by Schamp et al. (2004) and is not sufficiently captured by the homophily concept provided by organizational psychology (McPherson et al. 2001). It is important to disentangle the concept of ‘personal proximity’ from the idea of ‘clicks’ as the two concepts are related but are not exactly the same. Personal proximity only captures the similarity of partners regarding personality traits and characteristics as well as the resultant behaviors. The ‘click’ can emerge from personal proximity when partners touch on the ‘sweet spot’ of the continuum of personal proximity. In this respect, our empirical material improves our understanding of personal proximity and ‘clicks’. Whereas earlier definitions of personal proximity (notably Caniëls et al. 2014) also refer to the extent to which partners enjoy each other’s company, we find that this is actually an indication of the resultant ‘click’. To give an example, TUD3A-2 points to his most prosperous collaborations based on ‘clicking’ with people whom he “knows, trusts and respects” giving the essence of the definition of clicks as “a mutual feeling of acceptance, appreciation and interest in each other’s ideas” (Caniëls et al. 2014). In terms of the homophily-principle personal proximity is expressed through “similarity” and the click is the “connection bred” as a result thereof (McPherson et al. 2001: 415). We should note, however, that the homophily-principle does not hold entirely as clicks are most likely to develop at an above average rather than perfect degree of similarity between individuals. Complementarity of personalities rather than similarity is crucial. In other words, one could still loath the prospect of working with a clone of oneself. Perfect personal proximity might also be a liability to the outcome of collaborations. Despite various obvious benefits discussed in the Introduction, collaborations can be risky and costly. Collaborations can expose partners to opportunism and may complicate protection of intellectual property (e.g. Granovetter 1985; Williamson 1973, 2002). Perhaps this explains our findings that temporary geographical proximity rather than geographical proximity is sought after, as maintaining some form of geographical distance may help to diminish the likelihood of intellectual property leakage or opportunism among personally close collaborators.

Finally, our recommendations for management and policy emerge straightforwardly from our findings. As partners who do not ‘click’ tend to terminate collaborations, investments in collaborations as well as the knowledge created in their context are partly lost. Therefore, university management and research policy makers have a vested interest in taking personal proximity into account. Researchers seem to be cautious with collaborations lacking sufficient personal proximity. Sometimes this may be for good reasons, in other instances it might simply come down to rejecting persons with traits and characteristics that are unfamiliar but may in fact enrich collaborations by increasing the variety of insights or approaches. Therefore, management should certainly consider to invest in trainings to enable researchers to work together with people less like themselves. A diversity policy including researchers differing in ethnical background, gender and age may help to foster personal proximity between diverse kinds of researchers. In a sense, this means that researchers receive training that widens the margins within which they feel personally proximate to others, thereby overcoming objective personal distance by perceived personal proximity (see “Conclusions” section). Moreover, current research policy—such as the European Commission’s research funding programs under Horizon 2020 that require consortia to include partners from different sectors, such as academia, industry or civil society—fosters personal proximity.

## Conclusions

Recent empirical studies into the effect of proximity on collaborations have largely focused on geographical, cognitive, organizational, and social proximity. We add to the existing literature by investigating personal proximity in detail. We suggest that personal proximity can make or break collaborations between partners even if partners are sufficiently close regarding other dimensions of proximity. By investigating the role personal proximity plays for collaborations we answer to calls for analyses of individual agency (Tödtling and Trippl 2012) and of relationships between partners in networks (Rutten and Boekema 2012). Moreover, with our study we acknowledge the fact that academic engagement activities crucially depend on individual-level decisions (Perkmann et al. 2013).

In line with the theoretical concept provided by Caniëls et al. (2014), our core result shows that personal proximity affects collaborative choices and processes in three ways. First, personal proximity enables collaborations as it inspires partners to select others with whom they ‘click’ and helps them to carry out joint activities, to produce output and to decide whether or not to proceed with a collaboration. Second, we show that personal proximity mediates the effect of both social proximity and temporary geographical proximity on collaborator selection in particular. Both social and temporary geographical proximity serve as vehicles to explore, assess, and develop personal proximity rather than directly affect collaborator selection. Third, whereas personal proximity serves as an enabler of collaborations, having the right combination of cognitive and organizational proximity is important for successful collaborations.

We suggest that our findings are rather under- than overestimating the effects of personal proximity. Academic researchers work in a world of reason and logic. Therefore, it is much easier for them to acknowledge the role of ‘objective factors’, such as cognitive, organizational, social and temporary geographical proximity. Hence, we suggest that academic researchers may be prone to rationalize their own behavior and may not want to fully admit that they include soft factors—personal likes and dislikes—in their decisions about collaborations.

As our findings on personal and related kinds of proximity are relevant beyond the particular collaborations we investigated, we suggest three avenues for further research. First, the concept of personal proximity can help to explain dynamics at higher levels than that of individual dyadic relationships (e.g. evolution of network structures and regions). Researchers work, collaborate and shape the dynamics of networks, i.e. the combination of the relatively stable institutions such as laws and the agency of individuals and key players such as firms and universities (Tödtling and Trippl 2012). To date we still know very little about the influence of personal features on the formation of collaborations and research networks as a whole. For example, personal proximity between some actors and policy makers may induce practices of playing favorites, which in turn affects the evolution of networks.

Second, the results of this study are based on eighteen interviews with leading Dutch nanotechnology researchers. It would be interesting to study how different dimensions of proximity affect dynamics in research collaborations of more junior researchers and researchers in different network positions (i.e., researchers outside of the largest connected component or researchers at the global top or bottom in terms of centrality). We suggest that more central researchers are most likely best able to ‘use’ personal and other kinds of proximity in their relationships to full effect. Furthermore, future research may want to benchmark our results to different cohorts of researchers in different knowledge fields.

Third, for future works it is important to note that our study measures *perceived* proximity (Wilson et al. 2008) rather than more objective similarity of individuals. One may argue that perceived closeness on the personal level is more interesting than objective similarity on the personal level, because the results illustrate that perception rather than actual similarity drives behavioral choices. In any case, future studies may aspire to adopt methodologies that allow for quantitative measurement of objective personal proximity—true homophily—and compare its relevance to perceived personal proximity. We encourage researchers to explore ways in which to operationalize objective personal proximity. The refinement of indicators for all proximity measures continues to be an essential area of improvement for the proximity literature. The extant literature relies largely on archival research and proxies that simplify constructs considerably. In this respect, the development of survey measurement instruments, recently undertaken by Heringa et al. (2014) complementing common archival research methods could be a productive avenue for future research. Measurement scales for personal proximity could either choose to use the ‘click’ as a proxy, such as Casciaro and Lobo (2008) measure personal liking with a single item, or choose to develop and test more refined instruments transferred from organizational psychology studies (e.g. Hogan and Holland 2003). These efforts would eventually also address questions regarding the optimal level of personal proximity and clarify to what extent divergent personalities can still complement each other.

## Appendix

See Tables 6 and 7

**Table 6:** Most influential researchers in the global nanotechnology research network

Location	Eigenvector centrality	Closeness centrality	Degree centrality	Eccentricity
United States	0.11180	0.18	5.15 × 10^−4^	15
Germany	0.10930	0.18	4.53 × 10^−4^	15
United States	0.10790	0.18	4.56 × 10^−4^	15
Germany	0.10611	0.18	4.10 × 10^−4^	15
Germany	0.10609	0.18	4.08 × 10^−4^	15

According to Eigenvector centrality

**Table 7:** Least influential researchers in the global nanotechnology research network

Location	Eigenvector centrality	Closeness centrality	Degree centrality	Eccentricity
China	8.92 × 10^−17^	0.07	1.84 × 10^−6^	22
Malaysia	2.12 × 10^−16^	0.07	3.68 × 10^−6^	22
Malaysia	2.13 × 10^−16^	0.07	3.68 × 10^−6^	22
India	2.52 × 10^−16^	0.08	1.84 × 10^−6^	20
Russia	4.19 × 10^−16^	0.08	5.52 × 10^−6^	21

According to Eigenvector centrality

## Footnotes

1 There is another Dutch university of technology situated in Wageningen. It specializes in agriculture and has less of a presence in Dutch nanotechnology research. This is also reflected in the fact that Delft University of Technology, Eindhoven University of Technology and University of Twente have formed the 3TU network (3TU 2014) that has played a crucial role in the context of nanotechnology.
